# Identifying Objective EEG Based Markers of Linear Vection in Depth

**DOI:** 10.3389/fpsyg.2016.01205

**Published:** 2016-08-10

**Authors:** Stephen Palmisano, Robert J. Barry, Frances M. De Blasio, Jack S. Fogarty

**Affiliations:** ^1^Centre for Psychophysics, Psychophysiology, and Psychopharmacology, University of WollongongWollongong, NSW, Australia; ^2^School of Psychology, University of WollongongWollongong, NSW, Australia; ^3^Brain & Behaviour Research Institute, School of Psychology, University of WollongongWollongong, NSW, Australia

**Keywords:** vection, self-motion perception, optic flow processing, EEG, psychophysiology, ERSP

## Abstract

This proof-of-concept study investigated whether a time-frequency EEG approach could be used to examine vection (i.e., illusions of self-motion). In the main experiment, we compared the event-related spectral perturbation (ERSP) data of 10 observers during and directly after repeated exposures to two different types of optic flow display (each was 35° wide by 29° high and provided 20 s of motion stimulation). Displays consisted of either a vection display (which simulated constant velocity forward self-motion in depth) or a control display (a spatially scrambled version of the vection display). ERSP data were decomposed using time-frequency Principal Components Analysis (t–f PCA). We found an increase in 10 Hz alpha activity, peaking some 14 s after display motion commenced, which was positively associated with stronger vection ratings. This followed decreases in beta activity, and was also followed by a decrease in delta activity; these decreases in EEG amplitudes were negatively related to the intensity of the vection experience. After display motion ceased, a series of increases in the alpha band also correlated with vection intensity, and appear to reflect vection- and/or motion-aftereffects, as well as later cognitive preparation for reporting the strength of the vection experience. Overall, these findings provide support for the notion that EEG can be used to provide objective markers of changes in both vection status (i.e., “vection/no vection”) and vection strength.

## Introduction

As we move through the world, our self-motions are registered by a variety of senses, including vision, audition, the vestibular system of the inner ear, the somatosensory system of cutaneous receptors, and the proprioceptive system of muscle and joint receptors (Dichgans and Brandt, 1978; Palmisano et al., 2011). While the appropriate stimulation of any of these sensory systems can generate a perception of self-motion, vision appears to play an especially important role (e.g., Palmisano et al., 2011). Typically our self-motions are accompanied by a particular type of visual motion stimulation known as global optical flow (Gibson, 1966). This global optical flow is capable of inducing compelling visual illusions of self-motion in physically stationary observers—commonly referred to as “vection” (“circular vection” refers to illusory self-rotations and “linear vection” refers to illusory self-translations—see Dichgans and Brandt, 1978; Palmisano et al., 2015)^1^. These vection illusions can be experienced in everyday life (e.g., when one is in a car that has stopped at a traffic light and an adjacent car rolls forward). They are now also common experiences in many modern computer graphics and virtual reality simulations (such as driving/flight simulators as well as a wide variety of video games—see Riecke, 2011).

While much of the critical research has yet to be done, it is often assumed that the neural processes underlying vection are closely related to those occurring during real self-motions (since vection and real self-motion both result in conscious subjective experiences of self-motion—see Brandt et al., 1998; Pitzalis et al., 2013; Palmisano et al., 2015). There is also emerging evidence that vection may have functional significance (see Palmisano et al., 2015 for a review). Vection appears important for navigation/spatial orientation (Riecke et al., 2015), and it might potentially aid in the transfer of training from simulation to real life (see Keshavarz et al., 2015; Palmisano et al., 2015). Unfortunately, difficulties obtaining reliable measures of vection have proved to be a major impediment to progress in both areas of research (Keshavarz et al., 2015; Palmisano et al., 2015). As Keshavarz et al. (2015) recently noted, “there are no well-validated, objective measures that can reliably identify or characterize the experience of vection” (p. 1). To date most research has relied on subjective self-report measures of vection, such as verbally (or otherwise) rating the nature of vection and pressing/releasing buttons to indicate the onset/offset of vection. As vection is by definition a subjective experience, such subjective responses would appear to be well-suited to measuring this experience. Indeed it is likely that even if alternative objective measures of vection could be identified, subjective measures would still be required to confirm the vection experience. However, these subjective self-report measures of vection are also thought to have important limitations. One problem is that the instructions and methods used, as well as the types of self-reports obtained, have varied markedly from study-to-study (e.g., different studies have rated vection in terms of its strength, its magnitude, its perceived speed, and its degree of saturation). This has made it difficult to compare vection data across studies. Another problem is that self-reported vection ratings can be susceptible to experimenter demands (such as if information biasing the observer toward reporting self-motion was present in their instructions) and observer cognitions (such as the observer's knowledge about the possibility for, or the impossibility of, physical self-motion) (e.g., Riecke, 2009; see Palmisano and Chan, 2004 for a discussion). It is also likely that, in at least some of these past studies, self-reported vection ratings were contaminated by confusion associated with unusual sensations experienced during the visual self-motion stimulation (such as feelings of uncertainty or instability, mild symptoms of motion, disorientation or motion sickness—Bonato et al., 2009). Self-reporting the vection time course (e.g., via button pressing) should be less susceptible to some of the above problems. However, it is still likely that self-reported vection onsets were inflated in many of the past studies. This vection onset inflation problem should be greatest early on, when the naïve observers first have to decide exactly what constitutes “vection” prior to responding, and would be exacerbated if subjects had not received sufficient vection practice prior to testing in the main experiment^2^.

The above issues are therefore drivers for researchers to identify/develop improved indicators of vection. In our 2015 vection review paper, we discussed several possible objective indicators of vection, including eye-movements, postural responses, and electroencephalography (EEG) (Palmisano et al., 2015). Keshavarz et al. (2015) have argued that EEG is a particularly promising candidate for an objective vection measure because it provides “temporally precise, online measures of the working brain,” “is portable,” “is inexpensive,” “easily administered,” “does not require an overt response from the participant,” “has a high temporal resolution,” and involves a “well-established” signal (p. 2). Also supporting the use of EEG as a vection measure, there is evidence that EEG can be used to discriminate vection and object motion perception. For example, Tokumaru et al. (1999) reported significant differences in EEG topography in the high alpha band during vection compared to viewing the same display when stationary (although there was not a common pattern in these differences across the five subjects tested). Another study by Thilo et al. (2003) measured visual evoked potentials (VEPs) during exposure to a large rotating pattern surrounding a smaller central checkerboard probe stimulus. They found that amplitudes of the first negative inflection (N70) to central probe stimulation were significantly reduced during roll vection (compared to object-motion perception) at sites Oz (midline occipital), O1 (left occipital), and O2 (right occipital). More recent research also suggests that EEG might be informative about the vection inducing potential of optic flow. While their 2.5–3.5 s stimulus exposures were too short to induce vection during EEG recording, Keshavarz and Berti (2014) found evidence that the N230 at O1 and O2 was more pronounced for stimuli that induce stronger vection during longer 45 s stimulus exposures (i.e., after the EEG recording session). Another recent study by Vilhelmsen et al. (2015) examined EEG during visually simulated forward self-motions at three different speeds (with static controls between each condition). They found that N2 peak latency increased and peak amplitude decreased in parietal channels P3 and P4 as the visually simulated speed increased (although it should be noted that they did not check for vection during their study). Taken together these findings suggest that EEG could be used to indicate cortical processing related to vection onset/offset as well as vection response scaling.

In order to search for such markers, one must first understand how vection is typically induced and how it is experienced perceptually. When a self-motion display is presented to a stationary observer there is a finite delay before any vection is reported (Brandt et al., 1973; Dichgans and Brandt, 1978; Bubka et al., 2008). The observer will first perceive object motion, then combined object-and-self-motion, and finally (assuming favorable/optimal vection conditions) exclusive self-motion—with vection strength generally building toward a plateau over time (e.g., Apthorp and Palmisano, 2014). The initial delay in experiencing vection is generally thought to reflect the time required for the observer to resolve the following sensory conflict: his/her visual input indicates self-motion, but the expected non-visual inputs (which would normally accompany the visually simulated self-motion) are absent (e.g., Zacharias and Young, 1981; Johnson et al., 1999). It is also worth noting that vection can also be experienced after all of the display motion has ceased. Prolonged viewing of a radially expanding pattern of optic flow is known to generate a strong contracting motion aftereffect. However, under the right circumstances, it can also produce a vection aftereffect that occurs in the opposite direction to the vection experienced during adaptation (e.g., Brandt et al., 1974; Seno et al., 2010, 2011).

In the present study, we recorded the EEG obtained both during and directly after exposure to typical vection (as well as motion control) displays. We then used the Event-related Spectral Perturbation (ERSP) to summarize the EEG changes associated with each of the visual motion stimuli tested. This is an established time-frequency approach that decomposes the EEG into its amplitude spectrum across the frequencies of interest at regular time intervals through the period surrounding each trial (see Barry, 2009; Barry et al., 2012). We decomposed these ERSPs via time-frequency Principal Components Analysis (see Barry et al., 2015). The application of these time-frequency methods in the vection field is novel.

## Materials and methods

The study consisted of a preliminary behavioral stimulus (i.e., vection check) experiment (*N* = 46) which was followed by the main EEG experiment (*N* = 10).

### Participants

This study was approved by the University of Wollongong Human Research Ethics Committee (HE12/422). The subjects were either 46 (aged *M* = 19.94, *SD* = 1.86 years; 23 males, 23 females) or 10 (aged *M* = 19.63, *SD* = 1.27 years; 3 males, 7 females) right-handed young adults. All of these subjects were naïve to the experimental hypotheses, had normal or corrected-to-normal vision, were clear of any visual or vestibular impairment, and presented no obvious signs of oculomotor or neurological pathology. They also reported no history of psychiatric illness, severe head injury, seizures, or psychoactive drug use, and confirmed abstinence from caffeine and tobacco for at least 2.5 h prior to testing. All gave written informed consent in line with a protocol approved by the joint University of Wollongong/South East Sydney and Illawarra Area Health Service Human Research Ethics Committee, in accordance with the Declaration of Helsinki.

### Visual stimuli

The visual motion stimuli were computer-generated “self-motion” and “motion control” displays that subtended a visual area 35° wide by 29° high (when viewed from the observer's vantage point). These displays were projected onto a screen^3^ located 2.2 m directly in front of the seated subject in an otherwise dark room. Both motion displays consisted of 1000 (moving) blue circular dots (each dot being 0.36° in diameter) presented on a black background. A stationary red fixation point was also superimposed onto the center of each frame^4^. The “self-motion” display (a radially expanding pattern of optic flow) simulated a constant velocity forward self-motion at 1 m/s through a 3D cloud of randomly positioned objects (Cloud dimensions were 30 m wide by 30 m high by 40 m deep—see Supplementary Movie 1). The “motion control” display was constructed by first dividing the “self-motion” display into six equally sized sectors (i.e., a 3 horizontal × 2 vertical grid) and then randomly scrambling the screen locations of these sectors (see Supplementary Movie 2 for the “scrambled-patch” motion control display). This scrambling produced globally incoherent motion, however, local motions were identical to those in the original (unscrambled) “self-motion” display. In addition to these two motion stimuli, a vertically oscillating vection display was also used as a reference stimulus for the subject's vection ratings. This vertically oscillating vection display was identical to the “self-motion” display, except that the position of the virtual camera was also oscillated up and down at a frequency of 2 Hz and an amplitude of 0.25 m. It was expected that this oscillating self-motion display would induce a more compelling vection experience than both the “self-motion” display and the “motion control” display (see Palmisano et al., 2008; see also Palmisano et al., 2011 for a review of the research into this oscillation advantage for vection). The choice of this “stronger” vection stimulus as a reference was deliberate. We wanted to reduce the likelihood of participants reporting vection when they were not actually experiencing it (e.g., due to possible experimental demands).

### Procedure

A preliminary behavioral stimulus (i.e., vection check) experiment was conducted prior to the main EEG experiment. Both experiments consisted of three separate blocks of 10 experimental trials. Each block presented five “self-motion” display trials and five “motion control” display trials, which were delivered in an order that was randomized across subjects.

**Figure 1:**
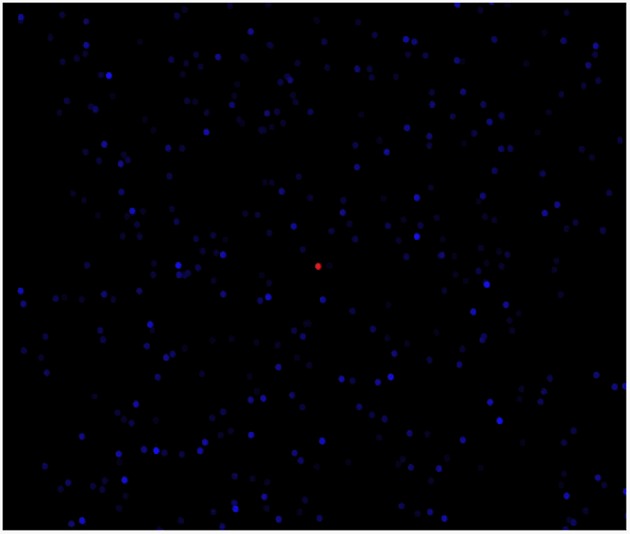
**The prestimulus fixation screen for self-motion**. This is the first frame of the self-motion display video, and included a red fixation point amidst the blue dots that radially expanded in the video clip.

**Figure 2:**
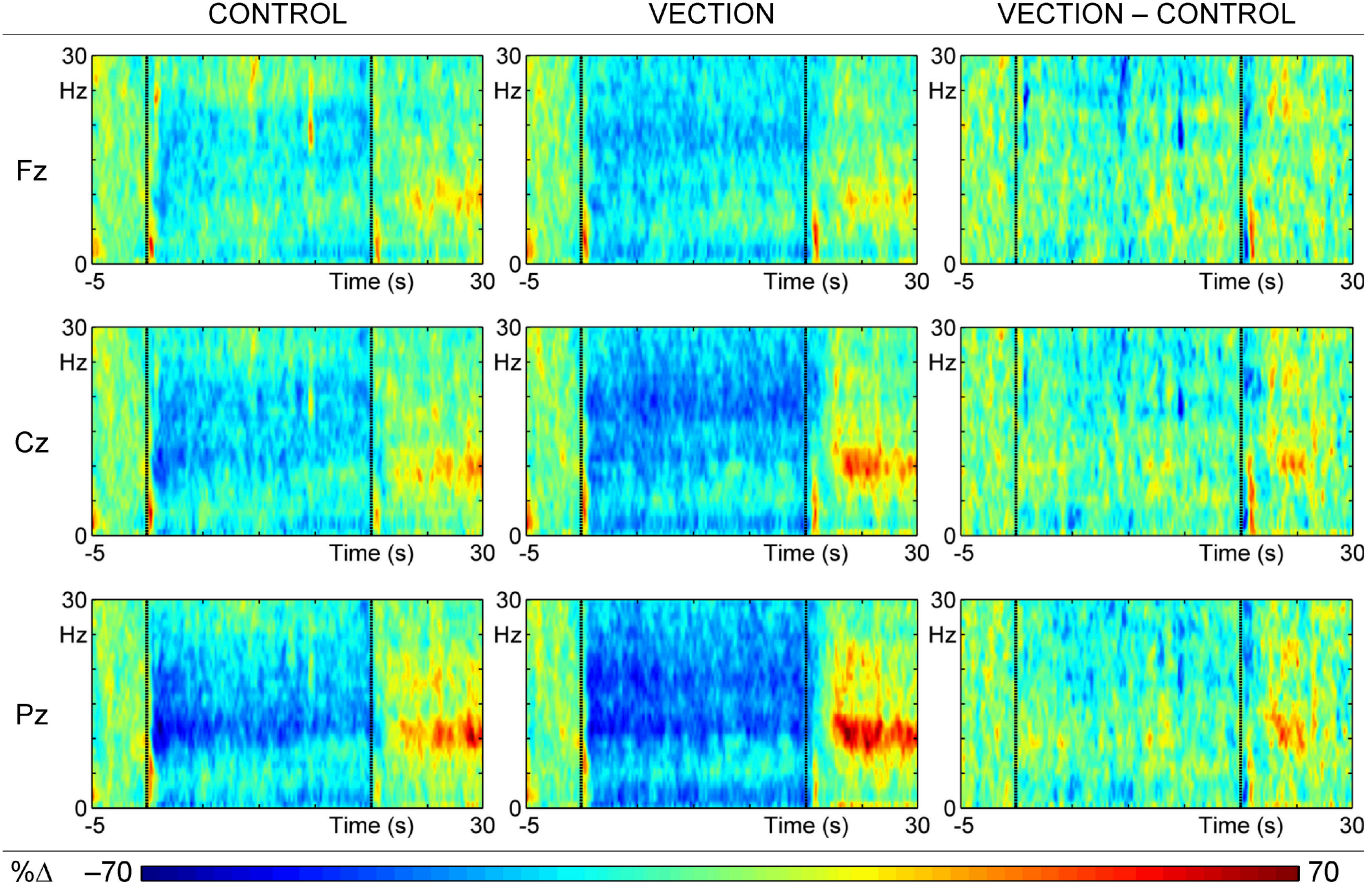
**ERSPs at each of the midline scalp sites (Fz, Cz, and Pz) for the “motion-control” condition (Control; left), the “self-motion” (Vection; center) condition, and their difference (Vection–Control; right)**. Each colored ERSP plot has three segments: baseline (from 5 s before motion to the beginning of motion), during motion (middle 20 s section of each plot), and after motion (last 10 s of plot); the visual display ceased at 30 s, when a tone signaled to report the intensity of the vection experience.

Prior to each of these blocks, subjects were presented with a single 20 s exposure to the vection reference display (i.e., the vertically oscillating pattern of radially expanding optic flow; they were re-exposed to this reference 4 more times during each block). The initial exposure to this standard stimulus was used to set the modulus for the subject's vection strength ratings (Stevens, 1957). After checking that this vertically oscillating reference stimulus had indeed induced vection, subjects were told to: (a) rate the strength of this vection experience as “50”; and (b) rate the strength of their vection experiences on subsequent trials in relation to this (e.g., a rating of “0” indicated that they felt stationary during that trial, whereas a rating of “25” indicated their vection experience was half as strong as during the vection reference). Trials were identical in both experiments (35 s visual exposure followed by verbal vection strength rating). Each trial consisted of the following 4 phases: (1) an initial 5 s exposure to the stationary pre-stimulus fixation screen (i.e., the first frame of the motion stimulus video clip—see Figure 1); (2) followed by 20 s exposure to the visual motion stimulus (either the “self-motion” or the “motion control” condition—see Supplementary Movies 1, 2 in Supplementary Materials); (3) followed by 10 s exposure to the stationary post-stimulus fixation screen (i.e., the final frame of the motion stimulus video clip); and finally, (4) an auditory tone (2000 Hz, 80 dB SPL, 50 ms + 15 ms rise/fall time) sounded to indicate the end of the exposure phase of the trial. This tone indicated that it was time for subjects to verbally report the strength of their vection experience for the motion phase of that trial.

Subjects minimized their eye-movements by focussing on the red fixation dot in the center of the screen during all four of the exposure phases of each trial. Prior to the main EEG experiment, subjects also completed a brief EOG calibration task. This required vertical and horizontal eye movements and eye blinks, so that the data could later be EOG corrected. EEG data in the main experiment were acquired using a Neuroscan Synamps 2 digital signal-processing system and Neuroscan 4.3.1 Acquire software. EEG data were recorded continuously from M2 and 28 scalp sites (Fp1, Fp2, F7, F3, Fz, F4, F8, FT7, FC3, FC4, FT8, T7, C3, Cz, C4, T8, TP7, CP3, CP4, TP8, P7, P3, Pz, P4, P8, O1, Oz, O2). An electrode cap with Ag/AgCl electrodes was referenced to M1, and grounded by an electrode located midway between Fp1/Fp2 and Fz. The data were recorded DC to 70 Hz with a 50 Hz notch filter. EOG was recorded using Ag/AgCl electrodes placed 2 cm above and below the left eye for vertical movements, and on the outer canthus of each eye for horizontal movements. Impedance was < 5 kΩ for cap, EOG, and reference electrodes. Scalp and EOG potentials were amplified with a gain of 500 and digitized at a rate of 1000 Hz.

### Data extraction

EEG data for the 10 subjects in the main experiment were first EOG corrected using the RAAA EOG Correction Program (Croft and Barry, 2000). Neuroscan 4.5 Edit software was then used to re-reference the data offline to the digital equivalent of linked mastoids, and band-pass filter the data (0.1–30 Hz, zero-phase shift, 24 dB/Octave), before single trial epochs were extracted for 5.15 s pre- to 30.15 s post-stimulus. The remaining data quantification was completed within MATLAB® (The Mathworks, Version 8.0.0.783, R2012b) using EEGLAB (Version 9.0.8.6b; Delorme and Makeig, 2004) and a modified version of the PCA functions made available by Kayser and Tenke (2003; http://psychophysiology.cpmc.columbia.edu/software/) in which the mean-correction of the loadings prior to component rotation is omitted, following Dien and Frishkoff (2005).

For each site for each trial of each subject, for a period from 5 s before display motion commenced to 10 s after this motion ceased, we decomposed the EEG signal in brief overlapping segments using the Fast Fourier Transform (FFT), and plotted the amplitudes of each frequency at the midpoint of each segment. Each such ERSP used sliding FFTs with a window size of 500 data points [zero padded to 1000 points (1 s duration)]; each was DC corrected, employed a 10% Hanning window, and yielded EEG magnitude data at 1 Hz resolution from 0 to 30 Hz. With data at 200 ms intervals, each ERSP was baselined across the 5 s pre-motion period; the baseline data was used to convert the whole ERSP to percentage change from baseline at each frequency and time point. This yielded a three-dimensional matrix of EEG amplitude at each frequency step and at each time point, containing all the information in the EEG throughout the trial; this contrasts with the loss of information in event-related potentials (ERPs) caused by averaging the within-subject responses over trials. These ERSPs were obtained for each trial of both “self-motion” and “motion control,” for each subject.

Rather than selecting arbitrary time and/or frequency bands to assess, we used an innovative time-frequency Principal Components Analysis (t–f PCA) to obtain a data-driven summary of important time/frequency hot spots in the ERSP data, following Barry et al. (2015). As in all PCAs, components are extracted in order of importance on the basis of the variance they carry; here, we arbitrarily ceased exploration when this figure reached a low of 0.1%. ERSP epochs from all sites were together subjected to temporal time-frequency PCA with Varimax rotation of all components. The rotation of all components yields an optimal distribution of error variance over the components rather than selecting a limited number of components for rotation (Kayser and Tenke, 2003). Input consisted of the data points in the time dimension for each of the 1 Hz frequency steps (175 times × 30 frequencies = 5250 variables), and 8400 cases (10 subjects × 2 conditions × 15 trials × 28 sites). In this process, the single trial amplitude data of each subject and site, over frequency and time, are concatenated into a single vector (over time), subjected to a temporal PCA, and all the resultant components are rotated to simple structure (Kayser and Tenke, 2003). Then the concatenation process is reversed for each component to obtain the three-dimensional ERSP structure; there is no difference in PCA outcome if the alternate concatenation over frequency is employed (Barry et al., 2015).

## Results

### Part A: behavioral vection check experiment

A preliminary behavioral vection check was first conducted on 46 subjects using the “self-motion” and spatially scrambled “motion control” displays (with a vertically oscillating version of the “self-motion display” used as a reference for the vection strength ratings). The strength of the vection induced by these “self-motion” and “motion control” displays was examined with a paired samples *t*-test. As expected the “self-motion” display produced significantly stronger verbal vection ratings (*M* = 48; *SD* = 14) than the “motion control” display (*M* = 35; *SD* = 19), *t*_(45)_ = −4.94, *p* < 0.001. This behavioral vection check demonstrated that (1) both types of visual display appeared to be capable of inducing some vection; and (2) the “self-motion” and “motion control” displays reliably induced vection with appreciably different strengths. From the original 46 subjects, we randomly selected a subset of 10 subjects for the main EEG experiment^5^.

### Part B: main EEG experiment

The 10 subjects each completed 15 “self-motion” trials and 15 “motion control” trials (resulting in 300 experimental trials in total). 20 s of motion stimulation was generally sufficient to induce vection in both conditions (vection was reported in 283 of the 300 experimental trials). There was no reported occurrence of motion sickness. No trials were lost through artifact, allowing all subjects to contribute complete EEG data sets to the ERSP computation.

#### Event-related spectral perturbation (ERSPs)

The mean ERSPs for each condition, and their difference, are shown in Figure 2 at the major midline sites. Each is a plot of the intensity of the relative change from pre-motion levels at each frequency (1 to 30 Hz) at 200 ms intervals through the 35 s epoch, averaged over trials and subjects (5250 data points at each site).

There is relatively little change over time in the EEG activity of the 5 s pre-motion baseline period, apart from a brief frontocentral delta event-related synchronization (ERS), marked by orange color, immediately after trial onset. Motion onset is marked by a similar brief delta-theta ERS, which is repeated just after motion ceases at 20 s. These three low frequency effects appear similar in all topography panels for both “self-motion” (i.e., Vection) and “motion control” (i.e., Control) display conditions, and most likely mark responses to display onset and motion onset/offset. In addition, extensive event-related desynchronization (ERD), marked by blue color, over a wide frequency range (particularly in alpha and beta) continues through the motion period, starting after the initial low frequency motion-onset ERS and continuing to the onset of the ERS after the end of the stimulus motion. The difference in relative EEG activity between the two display conditions (i.e., Vection—Control), shown in the right panel of Figure 2, confirms that the greater desynchronization for “self-motion” displays is most apparent in the beta band, suggesting that the alpha band desynchronization reflects a common response to visual stimulation in both conditions. After motion offset, the most dominant feature of the ERSPs is a series of ERSs in the alpha band, with a posterior dominance, that appear greater for the “self-motion” than the “motion control” conditions. However, the late alpha synchronization, just prior to the end of the trial, appears to have been canceled in the difference ERSP, suggesting that it also may reflect some common activity rather than being related to the differential vection experience. These “self-motion” and “motion control” data were input to a temporal t–f PCA.

#### Time-frequency principal component analysis (t–f PCA)

For the t–f PCA of these ERSPs, unrestricted Varimax rotation (with a total of 5250 components) yielded 52 components that carried from 4.60 down to 0.10% of the variance. Smaller components (with < 0.10% of the variance) are likely to be of little relative importance in understanding vection. These (0.99% of the components) accounted for a total of 33.07% variance, demonstrating substantial data summary and reduction. The output ERSP (at the peak channel) and time-frequency headmap data from each of the 52 retained components are summarized in Supplementary Table 1, and plotted in Supplementary Figure 1, in peak temporal order.

**Figure 3:**
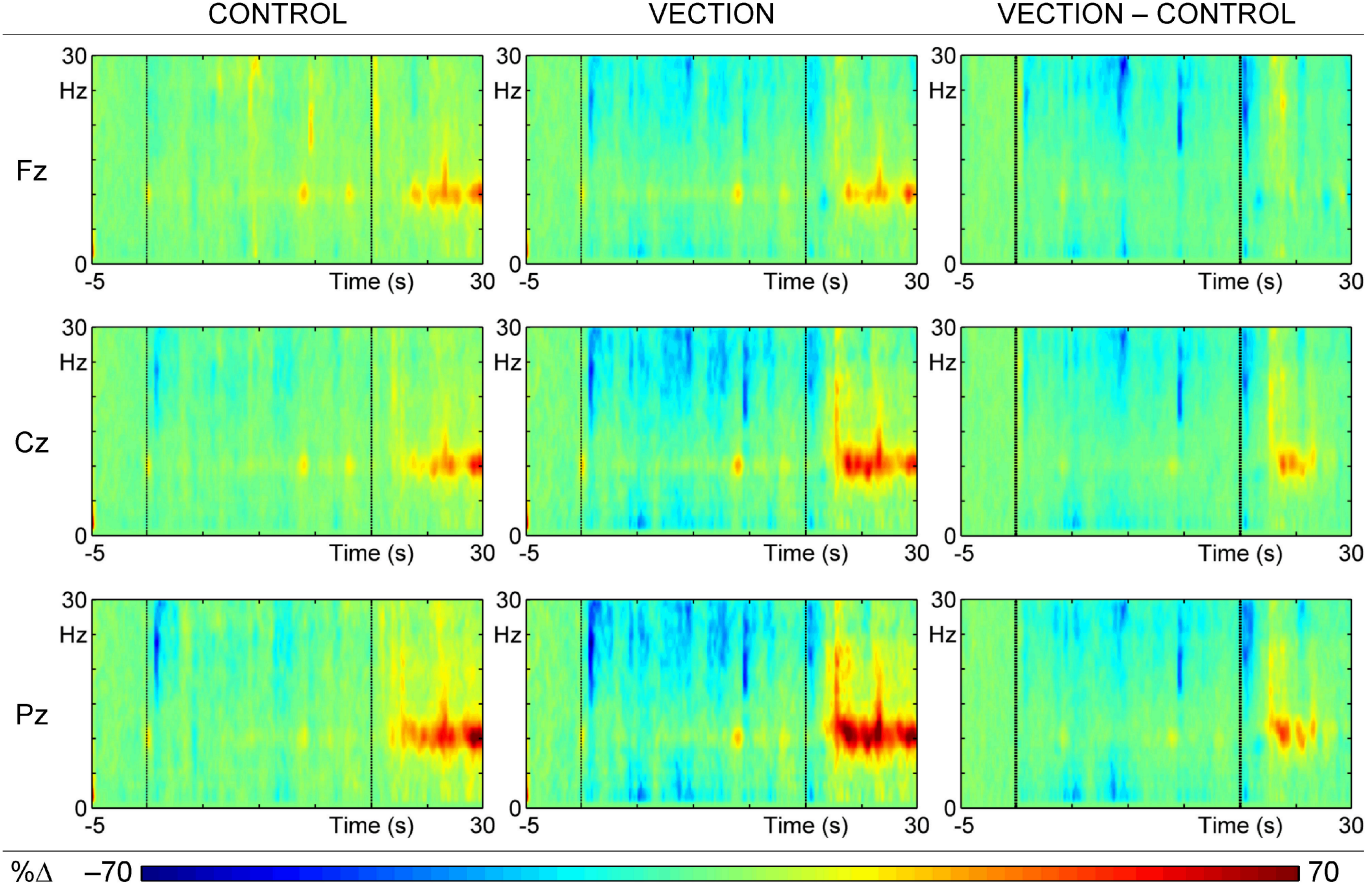
**ERSPs reconstituted from the sum of the first 52 t–f components of the PCA for the “motion-control” condition (Control; left), the “self-motion” condition (Vection; center), and the difference between these conditions (Vection–Control; right)**. Each colored ERSP plot has three segments: baseline (from 5 s before motion to the beginning of motion), during motion (middle 20 s section of each plot), and after motion (last 10 s of plot); the visual display ceased at 30 s, when a tone signaled to report the intensity of the vection experience.

Figure 3 shows the mean reconstituted ERSPs calculated from the sum of the selected time-frequency (t–f) components, for the “self-motion” and “motion control” displays, and their difference. Plots are averaged over trials and subjects at the major midline sites, corresponding to the raw/input ERSPs in Figure 2. Comparison with Figure 2 shows a reasonable fit between the raw and reconstituted ERSPs, apart from the loss of the extensive diffuse ERD (blue) across all sites and conditions, suggesting that this activity carried little systematic variance, and hence was not concentrated in the major PCA factors.

#### Components related to vection

This stage of the exploratory analyses used simple correlations across all 300 experimental trials (i.e., including both the “self-motion” and “motion control” trials for each subject). We correlated the individual reports of experienced vection strength, and the individual t–f component peak amplitudes for that trial. These correlations were carried out for all retained components. We regarded these as exploratory and no correction was made for multiple testing.

**Figure 4:**
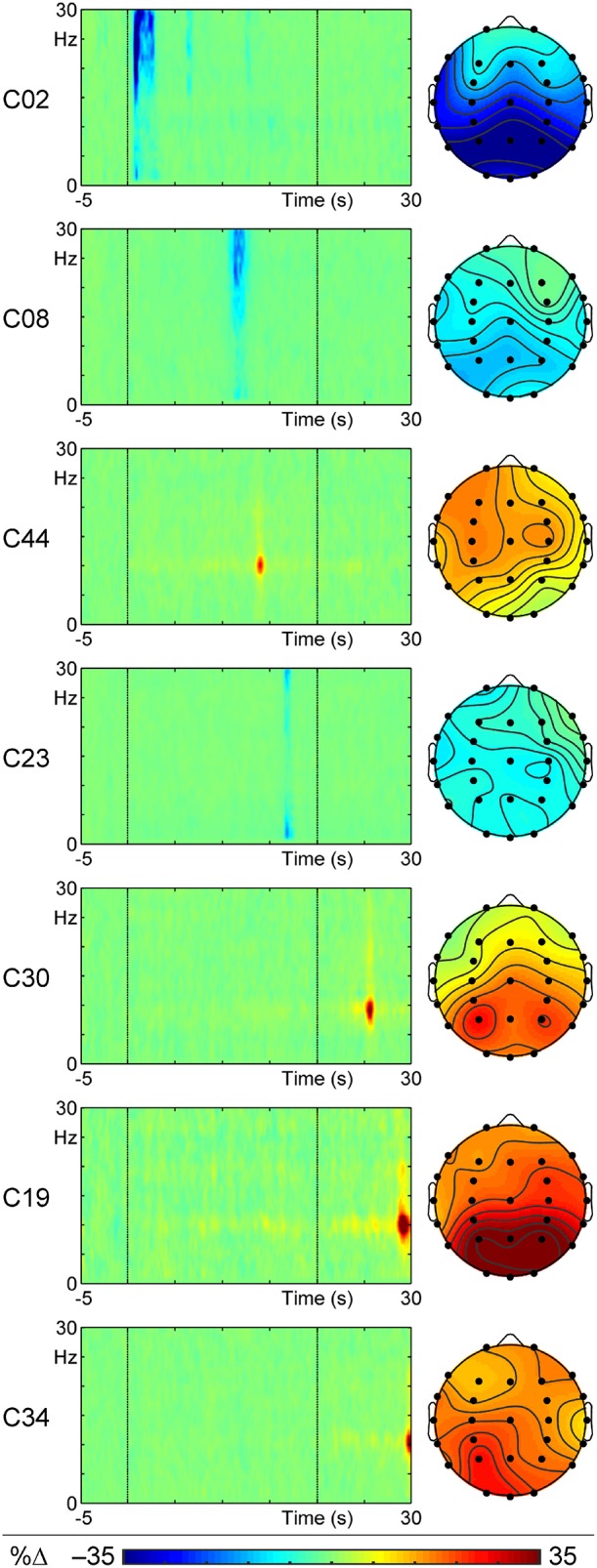
**ERSPs at the peak channel and headmaps at the peak frequency and latency for the seven t–f components that correlated with reported vection intensity, displayed in order of peak latency from motion onset**. The component number (which lists components in order of the % variance they carry) is shown for each on the left of the ERSP.

Of the 52 t–f components examined, seven correlated significantly with the intensity of the reported vection experience. These are listed in Table 1 and shown in Figure 4 in order of increasing onset latency of the component, and described in temporal sequence below. Four of these components (**C02**, **C08**, **C44**, **C23**) were observed during the actual stimulus motion, whereas the last three components (**C30**, **C19**, **C34**) occurred after the stimulus motion ceased.

**Table 1:** **Summaries for the components related to vection intensity, in peak temporal order**.

**Component**		**C02**	**C08**	**C44**	**C23**	**C30**	**C19**	**C34**
Peak	Amplitude (%Δ)	−39.27	−14.09	16.95	−10.65	25.79	41.46	25.25
	Frequency (Hz)	24	23	10	1	9	10	10
	Latency (s)	0.9	11.3	13.9	16.9	25.7	29.1	29.9
	Channel	Pz	P7	C3	FT7	P3	P4	O1
Frequency range (Hz)		18−27	21−30	9−11	1−30	8−10	9−11	9−11
Latency range (s)	t1	0.7	11.3	13.7	16.7	25.3	28.9	29.7
	t2	0.9	11.9	14.3	17.3	25.9	29.5	29.9
Variance (%)		4.26	1.20	0.12	0.28	0.18	0.34	0.16

*Underlined components are negative at their peak. Shaded components occur during motion. t1, component onset; t2, component offset*.

The first of these vection-related components, **C02** (a beta ERD), occurred between 0.7 and 0.9 s after stimulus motion began, and was maximal 0.9 s from motion onset. This component was a decrement in amplitude (shown as the blue ERD in Figure 4) that spanned 18–27 Hz, which was dominant at 24 Hz. Lower amplitude elements extended up to 30 Hz (with a minor delta ERD at 1 Hz) and to ~2.5 s and had recurrent small peaks at ~6.5 and 13 s after stimulus motion onset. The t–f headmap in Figure 4 shows this beta ERD to be dominant in the parietal region. This beta ERD decreased with increasing intensity of the reported vection experience (*r* = 0.122, *p* < 0.05).

The second vection-related component, **C08** (also a beta ERD), had a peak latency of 11.3 s, with a range from 11.3 to 11.9 s from the beginning of the stimulus motion. This component was another decrement in amplitude (shown as the blue ERD in Figure 4) that spanned 21–30 Hz, and was dominant at 23 Hz. Its t–f headmap in Figure 4 shows this decrement in intensity to be dominant in left parietal regions. Like **C02**, this beta ERD also decreased with increasing intensity of the reported vection experience (*r* = 0.116, *p* < 0.05).

The third vection-related component, **C44** (an alpha ERS) occurred between 13.7 to 14.3 s after stimulus motion began, and was maximal at 13.9 s from motion onset. **C44** was an increment (ERS) of 9 to 11 Hz alpha activity, with a peak frequency at 10 Hz, as shown in its peak channel ERSP plot in Figure 4. The t–f headmap in Figure 4 shows that this component had a diffuse left frontocentral distribution. This alpha ERS component increased with increasing intensity of the reported vection experience (*r* = 0.153, *p* < 0.01).

As shown in the peak channel ERSP plot of Figure 4, the fourth vection-related component, **C23**, was the last to occur during stimulus motion. It ranged in duration from 16.7 to 17.3 s from motion onset, peaking at 16.9 s. This was a delta ERD, with a dominant frequency of 1 Hz, but ranging up to 30 Hz (i.e., a wideband ERD with a peak in the delta band but activity increasing again in the high beta range). **C23** was diffuse, but with a reduction in right frontocentral regions (see its headmap in Figure 4), and its intensity decreased with increasing intensity of the reported vection (*r* = 0.113, *p* < 0.05).

The fifth vection-linked component, **C30**, occurred 5.3 to 5.9 s *after* stimulus motion offset, with a peak at 25.7 s from motion onset; see its peak channel ERSP plot in Figure 4. This component was an alpha ERS, ranging from 8 to 10 Hz, with maximum intensity at 9 Hz. Its t–f headmap in Figure 4 shows the ERS to be maximal in parietal regions with a left enhancement. This component increased with increasing vection reports (*r* = 0.120, *p* < 0.05).

**C19**, the next vection-linked component, ranged from 8.9 to 9.5 s *after* motion offset, with its maximum peak at 9.1 s (see peak channel ERSP in Figure 4). This was an alpha ERS, with frequency ranging from 9 to 11 Hz (maximum at 10 Hz). The t–f headmap in Figure 4 shows it to have a parieto-occipital distribution. This ERS decreased with increasing vection experience (*r* = –0.119, *p* < 0.05).

This was closely followed by the last vection-related component, **C34**. The peak channel ERSP plot for **C34** in Figure 4 shows that it occurred at the end of the epoch, ranging from 29.7 to 29.9 s (maximum at 29.9 s). C34 was another alpha ERS, with frequencies ranging from 9 to 11 Hz, and a maximum at 10 Hz; it had a diffuse distribution with a maximum in left posterior regions (see its headmap in Figure 4). This alpha ERS also decreased with increasing vection experience reported (*r* = −0.106, *p* < 0.05).

## Discussion

Stability of the relative ERSPs in the pre-motion period indicates that there was little anticipatory brain activity prior to motion onset. In addition, the similar brief delta-theta synchronizations shown at trial onset, motion onset, and motion offset are expected markers of the trial/display onset and motion onset/offset, such as would be reflected in the ERPs time-locked to these events. Further, the parietal (Pz) alpha desynchronization throughout the motion period, apparent in the left and center panels of Figure 2, essentially disappeared in the difference ERSP (right panel), suggesting that this reflects the expected alpha response to visual stimulation.

Although this parietal alpha desynchronization appears to have been consistent across conditions, neither it nor the diffuse ERDs apparent in the non-alpha bands during motion (see the center panel of Figure 2) emerged as major components in the t–f PCA. This likely reflects the low variance associated with long-lasting activity in a particular frequency band and topography (the parietally-dominant alpha desynchronization associated with visual stimulation) or low variance associated with noise in the other bands varying randomly in time, frequency, and topography.

The major finding here was that our time-frequency Principal Component Analysis (t–f PCA) revealed seven t–f components that correlated significantly with the reported strength of the vection experience—some or all of these components may form the basis of EEG markers of the strength and/or time course of vection. Four of these t–f components were found to peak during the actual visual stimulus motion (**C02**, **C08**, **C44**, and **C23**). After display motion ceased, three further t–f components were also found to be related to the reported vection experience (**C30**, **C19**, and **C34**—all ERSs in the alpha band).

As was noted earlier, this was primarily a proof of concept study. However, based on past behavioral vection data one can speculate about what these different t–f components might represent. We will start by discussing the t–f components found to peak during the actual visual motion stimulation (potential real-time markers of vection presence/absence as well as indicators of vection strength).

Given its occurrence so soon after stimulus motion onset (i.e., peaking ~1 s after), and its broad posterior topography (including occipital, parietal, and temporal regions of the cortex), the earliest **C02** component might reflect pre-vection activity (see also Keshavarz and Berti, 2014). To perceive radial optic flow as self-motion, one first needs to inhibit the default visual processing responsible for object/scene motion perception. So it is possible that this beta ERD reflects the inhibition of normal visual object-motion processing prior to actual vection induction.

Another beta ERD, **C08**, was also observed later during the motion period, peaking ~11 s after stimulus motion onset. Given its much later timing and its (primarily left parietal) topography, it is possible that **C08** reflects either the onset of vection or directly precedes the experience of illusory self-motion. If such speculations are valid, then **C44** (an alpha ERS peaking ~14 s after stimulus motion onset) might indicate the subject's conscious decision that he/she was experiencing vection—as it occurred primarily in the frontocentral region (implicated in executive functioning) and was also the only t–f component during stimulus motion to be positively related to vection intensity. There are of course other possibilities. For example, **C44** might instead indicate preparatory motor cortex activity aimed at compensating for the visually perceived self-motion.

Given its timing, the last t–f component observed during the motion period, **C23** (a delta ERD), could potentially represent exclusive/peak vection. For example, it is possible that this late inhibitory activity further reduced/extinguished any lingering perceptions of object motion—with the result being that all (not just some) of the optic flow was perceived to be due to self-motion. As with the other possibilities outlined above, this hypothesis of course needs to be tested/confirmed in future studies.

As noted above, after display motion ceased, three further t–f components were also found to be related to the reported vection experience (these were all ERSs in the alpha band). The first alpha synchronization **(C30)** in this post-motion period was positively related to the rated vection experience. Previously, Seno et al. (2011) found that 20 s exposure to radially expanding optic flow generated vection aftereffects that lasted ~4 s (on average), as well as general motion aftereffects that lasted ~3 s (on average). Both types of aftereffect were experienced in the current study (confirmed by subjects during the debriefing session). Given that **C30** peaked ~5.7 s after display motion offset, it might reflect the processing associated with either type of aftereffect. However, since the intensity of **C30** was positively related to vection strength in the current study, and vection aftereffects (but not motion aftereffects) are known to increase with the strength of the vection experienced during adaptation (see Seno et al., 2010), we propose that **C30** is more likely to represent activity associated with vection aftereffects.

The two subsequent t–f components (**C19** and **C34**) were both negatively related to the rated vection intensity, suggesting that they might relate instead to cognitive activity in preparation for the report of the intensity of the vection experience required after this period—perhaps greater activity is required to characterize weaker vection. At present such interpretations are only speculative, but post-experimental enquiry may disentangle these effects in future studies.

The major problem in the EEG portion of this study was the small number of subjects (*N* = 10). This severely reduced the power of the study, and it clearly requires replication with more subjects in order to clarify and refine the present findings. Despite this profound limitation, the study serves to demonstrate the viability of the time-frequency ERSP approach to investigate the EEG correlates of the vection experience, and the use of t–f PCA to decompose the ERSP into a smaller number of components that can be linked to the intensity of the vection experience.

Another potential limitation of this study was the relatively small size of our visual displays (only 35° wide by 29° high). While this FOV was able to reliably induce vection with our radial flow “self-motion” displays (also demonstrated previously by Andersen and Braunstein, 1985), and we made sure to anchor our vection ratings with a known strong vection stimulus (vertically oscillating radial flow), future vection studies should ideally utilize larger visual stimulus displays.

## Conclusions

We demonstrate that it is possible to record EEG during the vection experience, and have—for the first time—linked the observed physiological changes to the rated intensity of the vection experience. As such, this study provides a proof of concept in relation to the methodology. Future studies with greater participant numbers will overcome the major limitation here and should provide robust information on the brain correlates of vection.

## Author contributions

All of the authors named above meet the criteria for authorship as they have: (1) made substantial contributions to the conception or design of the work; or the acquisition, analysis, or interpretation of data for the work; (2) been involved in drafting the work for important intellectual content; (3) provided final approval of the version to be submitted for review; and (4) agreed to be accountable for all aspects of the work in ensuring that questions related to the accuracy or integrity of any part of the work are appropriately investigated and resolved.

### Conflict of interest statement

The authors declare that the research was conducted in the absence of any commercial or financial relationships that could be construed as a potential conflict of interest.

## Footnotes

^1^It should be noted that vection is increasingly used to refer to other self-motion experiences, including non-visual illusions of self-motion (such as auditory-induced vection—see Väljamäe, 2009) as well as visually-mediated self-motion perception during multisensory stimulation (e.g., Palmisano et al., 2014). Please see Palmisano et al. (2015) for a review of the use of this terminology.

^2^One reviewer noted that some vection studies either did not report or failed to include any vection training.

^3^The physical edges of this screen could not be seen. In addition, we created a penumbra around the edges of the display to obscure any possible sharp CGI edges.

^4^Past eye-tracking research has shown that this type of fixation point allows subjects to hold their eyes close to stationary during conditions very similar to the radial flow displays tested in this study (both the vertically oscillating and purely radial displays—see Palmisano et al., 2012). Any (minimal) residual eye movements would have been corrected by the EOG correction program, which would have removed their direct ocular effects from the EEG.

^5^An independent samples t-test confirmed that the vection ratings for this EEG group (*M* = 41.3) were not significantly different to those for the Non-EEG group (*M* = 42.92), *t*_(43)_ = −0.31, *p* > 0.05.

## Supplementary material

Supplementary Figure 1 **Peak channel ERSPs and peak time-frequency headmaps for the first 52 t–f components extracted, displayed in order of peak latency**. The component number (which lists components in order of the % variance they carry) is shown for each on the left of the ERSP.

Supplementary Table 1 **Summaries for the largest 52 components, in peak temporal order**. Components are presented in groups of 13 to save space. Underlined components are negative at their peak. t1, component onset; t2, component offset.

Supplementary Movie 1 **“Self-motion” display**. This simulated constant velocity forwards self-translation at 1 m/s. This is a 5 s section of a 20 s display.

Supplementary Movie 2 **“Motion control” display**. This was constructed by first dividing the “self-motion” display into six equally sized sectors and then randomly scrambling the screen locations of these sectors. This is a 5 s section of a 20 s display.
