# Supplementary figures and images for: Identifying Objective EEG Based Markers of Linear Vection in Depth
Source: Front Psychol. 2016 Aug 10;7:1205. doi: 10.3389/fpsyg.2016.01205 (PMC4979253; doi:10.3389/fpsyg.2016.01205)

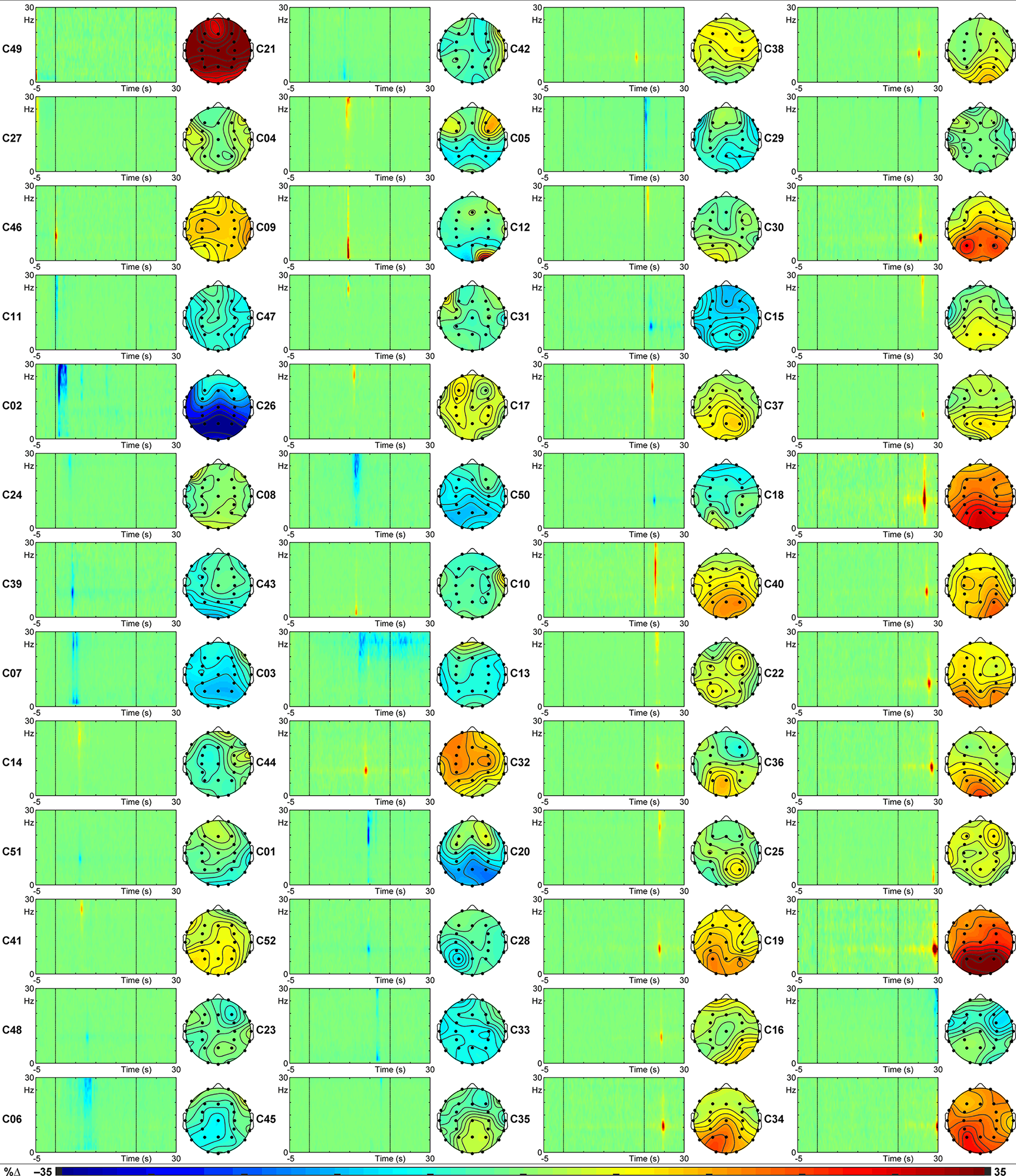

Supplement: Supplementary Figure 1 — Peak channel ERSPs and peak time-frequency headmaps for the first 52 t–f components extracted, displayed in order of peak latency. The component number (which lists components in order of the % variance they carry) is shown for each on the left of the ERSP. [file Image1.TIF]
