# Supplementary material for: Identifying Objective EEG Based Markers of Linear Vection in Depth
Source: Front Psychol. 2016 Aug 10;7:1205. doi: 10.3389/fpsyg.2016.01205 (PMC4979253; doi:10.3389/fpsyg.2016.01205)
Supplement: Supplementary Table 1 — Summaries for the largest 52 components, in peak temporal order. Components are presented in groups of 13 to save space. Underlined components are negative at their peak. t1, component onset; t2, component offset. [file Table1.PDF]

**Supplementary Table 1** Summaries for the largest 52 components, in peak temporal order.

| ***Component (1-13)*** | | C49 | C27 | C46 | C11 | C02 | C24 | C39 | C07 | C14 | C51 | C41 | C48 | C06 |
|---|---|---|---|---|---|---|---|---|---|---|---|---|---|---|
| Peak | Amplitude (%Δ) | 44.34 | 6.96 | 16.89 | -10.30 | -39.27 | -5.99 | -15.34 | -14.28 | 6.53 | -8.20 | 10.08 | -7.06 | -9.74 |
| | Frequency (Hz) | 1 | 24 | 10 | 25 | 24 | 26 | 10 | 1 | 24 | 10 | 26 | 10 | 30 |
| | Latency (ms) | -4900 | -4500 | 100 | 300 | 900 | 3500 | 4100 | 5100 | 5900 | 6100 | 6500 | 7900 | 8500 |
| | Channel | C4 | FT7 | TP8 | TP7 | Pz | FT7 | Oz | P4 | FT8 | Oz | P7 | F4 | Cz |
| Frequency range (Hz) | | 1-2 | 20-30 | 9-11 | 16-30 | 18-27 | 25-30 | 8-11 | 1-28 | 21-30 | 9-11 | 25-28 | 9-11 | 20-30 |
| Latency range (ms) | t1 | -4900 | -4700 | -100 | 100 | 700 | 3300 | 4100 | 4300 | 5700 | 5900 | 6300 | 7700 | 7100 |
| | t2 | -4900 | -4500 | 300 | 500 | 900 | 3700 | 4300 | 5500 | 6100 | 6300 | 6700 | 8100 | 8900 |
| Variance (%) | | 0.10 | 0.21 | 0.11 | 0.59 | 4.26 | 0.27 | 0.15 | 1.27 | 0.48 | 0.10 | 0.13 | 0.11 | 2.16 |

| ***Component (14-26)*** | | C21 | C04 | C09 | C47 | C26 | C08 | C43 | C03 | C44 | C01 | C52 | C23 | C45 |
|---|---|---|---|---|---|---|---|---|---|---|---|---|---|---|
| Peak | Amplitude (%Δ) | -7.93 | 13.95 | 39.09 | 10.55 | 10.87 | -14.09 | 11.62 | -11.44 | 16.95 | -17.86 | -11.60 | -10.65 | -5.48 |
| | Frequency (Hz) | 2 | 29 | 1 | 25 | 26 | 23 | 1 | 22 | 10 | 18 | 10 | 1 | 30 |
| | Latency (ms) | 8700 | 9500 | 9700 | 9700 | 11100 | 11300 | 11500 | 12500 | 13900 | 14700 | 14700 | 16900 | 17700 |
| | Channel | FP2 | FC4 | O2 | F7 | F3 | P7 | FT8 | TP7 | C3 | P4 | P3 | FT7 | T8 |
| Frequency range (Hz) | | 1-5 | 28-30 | 1-3 | 23-26 | 24-28 | 21-30 | 1-4 | 21-30 | 9-11 | 17-27 | 9-11 | 1-30 | 28-30 |
| Latency range (ms) | t1 | 8500 | 9300 | 9500 | 9500 | 10900 | 11300 | 11500 | 12300 | 13700 | 14500 | 14500 | 16700 | 17700 |
| | t2 | 8900 | 9900 | 9700 | 9700 | 11300 | 11900 | 11700 | 20100 | 14300 | 14700 | 14900 | 17300 | 17900 |
| Variance (%) | | 0.30 | 2.64 | 0.88 | 0.11 | 0.23 | 1.20 | 0.13 | 3.35 | 0.12 | 4.60 | 0.10 | 0.28 | 0.12 |

| ***Component (27-39)*** | | C42 | C05 | C12 | C31 | C17 | C50 | C10 | C13 | C32 | C20 | C28 | C33 | C35 |
|---|---|---|---|---|---|---|---|---|---|---|---|---|---|---|
| Peak | Amplitude (%Δ) | 9.40 | -13.33 | 7.96 | -14.54 | 11.15 | -12.89 | 16.60 | 7.63 | 13.08 | 9.60 | 17.08 | 11.72 | 21.61 |
| | Frequency (Hz) | 10 | 22 | 26 | 9 | 21 | 11 | 21 | 30 | 12 | 23 | 10 | 10 | 11 |
| | Latency (ms) | 18100 | 20500 | 20900 | 21700 | 22100 | 22500 | 22900 | 23100 | 23300 | 23900 | 23900 | 24300 | 24700 |
| | Channel | C4 | F7 | O1 | FP2 | P4 | FP1 | P4 | P3 | Pz | P4 | P3 | P8 | O1 |
| Frequency range (Hz) | | 9-11 | 1-30 | 19-30 | 8-10 | 18-30 | 10-12 | 13-30 | 23-30 | 11-13 | 21-27 | 9-11 | 9-12 | 9-12 |
| Latency range (ms) | t1 | 17900 | 20300 | 20700 | 21500 | 21900 | 22300 | 22700 | 22900 | 23100 | 23700 | 23700 | 24100 | 24500 |
| | t2 | 18300 | 20700 | 21100 | 22100 | 22300 | 22700 | 22900 | 23500 | 23700 | 24100 | 24100 | 24500 | 24900 |
| Variance (%) | | 0.13 | 2.41 | 0.55 | 0.17 | 0.37 | 0.10 | 0.63 | 0.53 | 0.17 | 0.33 | 0.20 | 0.17 | 0.16 |

| ***Component (40-52)*** | | C38 | C29 | C30 | C15 | C27 | C18 | C40 | C22 | C36 | C25 | C19 | C16 | C34 |
|---|---|---|---|---|---|---|---|---|---|---|---|---|---|---|
| Peak | Amplitude (%Δ) | 14.17 | -5.15 | 25.79 | 7.74 | 10.37 | 29.21 | 19.43 | 19.70 | 22.71 | 9.98 | 41.46 | -10.99 | 25.25 |
| | Frequency (Hz) | 11 | 29 | 9 | 28 | 10 | 12 | 10 | 9 | 11 | 3 | 10 | 29 | 10 |
| | Latency (ms) | 25300 | 25500 | 25700 | 26100 | 26100 | 26700 | 27100 | 27700 | 28500 | 28900 | 29100 | 29500 | 29900 |
| | Channel | O2 | FT8 | P3 | P4 | Pz | Oz | O2 | O1 | Oz | F4 | P4 | FC4 | O1 |
| Frequency range (Hz) | | 10-13 | 28-30 | 8-10 | 18-30 | 9-11 | 9-15 | 9-11 | 8-10 | 10-12 | 1-6 | 9-11 | 23-30 | 9-11 |
| Latency range (ms) | t1 | 25100 | 25300 | 25300 | 25900 | 25900 | 26500 | 26900 | 27500 | 28100 | 28700 | 28900 | 29300 | 29700 |
| | t2 | 25500 | 25700 | 25900 | 26300 | 26300 | 26700 | 27300 | 28100 | 28700 | 28900 | 29500 | 29700 | 29900 |
| Variance (%) | | 0.15 | 0.19 | 0.18 | 0.40 | 0.16 | 0.35 | 0.15 | 0.28 | 0.16 | 0.26 | 0.34 | 0.40 | 0.16 |

*Notes:* Components are presented in groups of 13 to save space. Underlined components are negative at their peak. t1 = component onset; t2 = component offset.
